# *Lrig2* and *Hpse2*, mutated in urofacial syndrome, pattern nerves in the urinary bladder

**DOI:** 10.1016/j.kint.2018.11.040

**Published:** 2019-05

**Authors:** Neil A. Roberts, Emma N. Hilton, Filipa M. Lopes, Subir Singh, Michael J. Randles, Natalie J. Gardiner, Karl Chopra, Riccardo Coletta, Zunera Bajwa, Robert J. Hall, Wyatt W. Yue, Franz Schaefer, Stefanie Weber, Roger Henriksson, Helen M. Stuart, Håkan Hedman, William G. Newman, Adrian S. Woolf

**Affiliations:** 1 Division of Cell Matrix Biology and Regenerative Medicine, School of Biological Sciences, Faculty of Biology Medicine and Health, University of Manchester, UK; 2 School of Allied Health Sciences, De Montfort University, Leicester, UK; 3 Division of Diabetes, Endocrinology and Gastroenterology, School of Medical Sciences, Faculty of Biology, Medicine and Health, University of Manchester, Manchester, UK; 4 Royal Manchester Children’s Hospital, Manchester University NHS Foundation Trust, Manchester Academic Health Science Centre, Manchester, UK; 5 Division of Evolution and Genomic Sciences, School of Biological Sciences, Faculty of Biology, Medicine and Health, University of Manchester, UK; 6 Manchester Centre for Genomic Medicine, St. Mary’s Hospital, Manchester University NHS Foundation Trust, Manchester Academic Health Science Centre, Manchester, UK; 7 Structural Genomics Consortium, Nuffield Department of Clinical Medicine, University of Oxford, UK; 8 Division of Pediatric Nephrology, Centre for Pediatric and Adolescent Medicine, University Hospital of Heidelberg, Im Neuenheimer Feld, Heidelberg, Germany; 9 Pediatric Nephrology, University-Children’s Hospital Marburg, Philipps-University Marburg, Germany; 10 Department of Radiation Sciences, Oncology, Umeå University, Umeå, Sweden; 11 Regional Cancer Center Stockholm/Gotland, Stockholm, Sweden

**Keywords:** autonomic, ganglia, gene, mouse, urination

## Abstract

Mutations in *leucine-rich-repeats and immunoglobulin-like-domains 2 (LRIG2)* or in *heparanase 2 (HPSE2)* cause urofacial syndrome, a devastating autosomal recessive disease of functional bladder outlet obstruction. It has been speculated that urofacial syndrome has a neural basis, but it is unknown whether defects in urinary bladder innervation are present. We hypothesized that urofacial syndrome features a peripheral neuropathy of the bladder. Mice with homozygous targeted *Lrig2* mutations had urinary defects resembling those found in urofacial syndrome. There was no anatomical blockage of the outflow tract, consistent with a functional bladder outlet obstruction. Transcriptome analysis revealed differential expression of 12 known transcripts in addition to *Lrig2*, including 8 with established roles in neurobiology. Mice with homozygous mutations in either *Lrig2* or *Hpse2* had increased nerve density within the body of the urinary bladder and decreased nerve density around the urinary outflow tract. In a sample of 155 children with chronic kidney disease and urinary symptoms, we discovered novel homozygous missense *LRIG2* variants that were predicted to be pathogenic in 2 individuals with non-syndromic bladder outlet obstruction. These observations provide evidence that a peripheral neuropathy is central to the pathobiology of functional bladder outlet obstruction in urofacial syndrome, and emphasize the importance of *LRIG2* and *heparanase 2* for nerve patterning in the urinary tract.

## Graphical abstract

**Figure:**
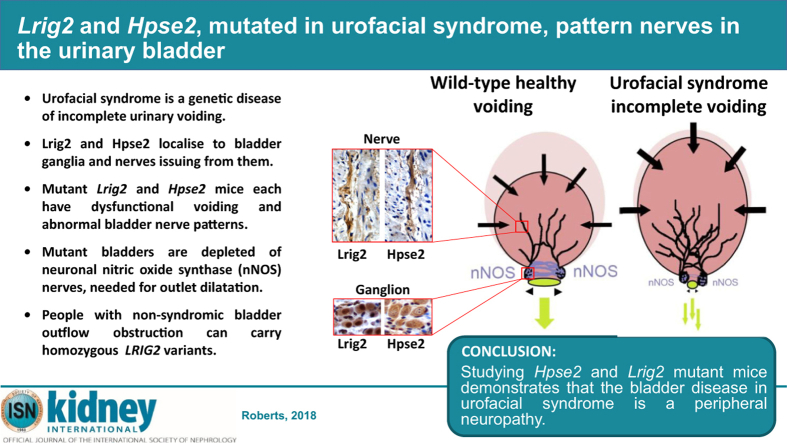

**Translational Statement**

These observations collectively provide evidence that a peripheral neuropathy is central to the pathobiology of urofacial syndrome bladder disease and emphasize the importance of leucine-rich repeats and Ig-like domains 2 and heparanase 2 in the urinary tract. Further definition of the molecular pathobiology underlying urofacial syndrome may reveal new therapeutic targets in bladder dysfunction. Gene therapy is now being used for certain rare genetic diseases. The encouraging clinical outcomes of babies with spinal muscular atrophy treated with adeno-associated virus–mediated replacement of a missing molecule^1^ could serve as a paradigm for congenital diseases of the urinary tract, such as urofacial syndrome. Here, a next step will be to test adeno-associated virus–mediated gene therapy in *heparanase 2* and *leucine-rich repeats and Ig-like domains 2* mutant mouse models of urofacial syndrome.

Bladders undergo filling and voiding cycles controlled by autonomic nerves.^2^, ^3^ Preganglionic autonomic neurons have cell bodies in the spinal cord, and their axons synapse within ganglia with postganglionic neurons whose axons innervate bladder muscles. Sympathetic noradrenergic neurons elicit outflow tract contraction during urinary storage, whereas during voiding, parasympathetic cholinergic motor neurons drive detrusor smooth muscle contraction and neuronal nitric oxide synthase (nNOS)–expressing neurons dilate the outflow tract.^4^ Urofacial, or Ochoa, syndrome (UFS) is an autosomal-recessive disease with around 150 families reported worldwide.^5^, ^6^ In persons with UFS, detrusor smooth muscle contracts against a poorly dilated outflow tract, causing functional bladder outlet obstruction.^5^, ^6^ This dyssynergia causes dribbling incontinence and residual urine, leading to a risk of ascending bacterial infection and kidney failure. The UFS bladder is a “non-neurogenic neurogenic bladder” resembling that caused by damaged bladder nerves, yet such gross lesions are absent in persons with UFS.^5^, ^6^ Persons with UFS typically have a facial expression “as if in pain or sadness when they tried to smile or laugh.”^5^ Ochoa,^5^ who first delineated the syndrome, and others^7^ speculated that there is a neuropathic basis for UFS.

Some families with UFS have biallelic variants in *heparanase 2* (*HPSE2*)^8^, ^9^, ^10^ encoding heparanase 2, an inhibitor of the enzyme activity of the classical heparanase protein,^11^ hereafter called “heparanase.” Homozygous *Hpse2* gene-trap mice have incomplete bladder emptying,^10^, ^12^ phenocopying UFS. Whether *Hpse2* mutant mouse bladders have innervation defects is unknown. Other families with UFS lack *HPSE2* mutations; some instead have biallelic variants of leucine-rich repeats and Ig-like domains 2 (*LRIG2*).^13^, ^14^ The most studied of the 3 mammalian Lrig proteins is Lrig1, a tumor suppressor that downregulates growth factor signaling.^15^, ^16^, ^17^ Belonging to the family of leucine-rich repeat–containing proteins, LRIG2 comprises multiple leucine-rich repeats, 3 Ig-like domains, a transmembrane segment, and a long cytosolic portion.^18^ Homozygous *Lrig2* mutant mice are protected from growth factor–induced gliomas, but their bladders were not studied.^19^

Because heparanase 2 and Lrig2 are immunodetected in fetal mouse^10^ and human^13^ bladder nerves, these proteins are well placed to affect nerve patterning. We hypothesized that UFS features a peripheral neuropathy of the bladder. We studied gene-targeted *Lrig2* mice, demonstrating that they have defective urination and abnormal patterns of bladder nerves. We went on to show that *Hpse2* mutant mice have similar defects. To date, only a few families with *LRIG2* mutations have been reported.^13^, ^14^ Accordingly, we studied persons with nonsyndromic bladder outlet obstruction and report homozygous *LRIG2* likely pathogenic missense variants in a subset.

## Results

### *Lrig2* mutant mice have abnormal urination

**Figure 1:**
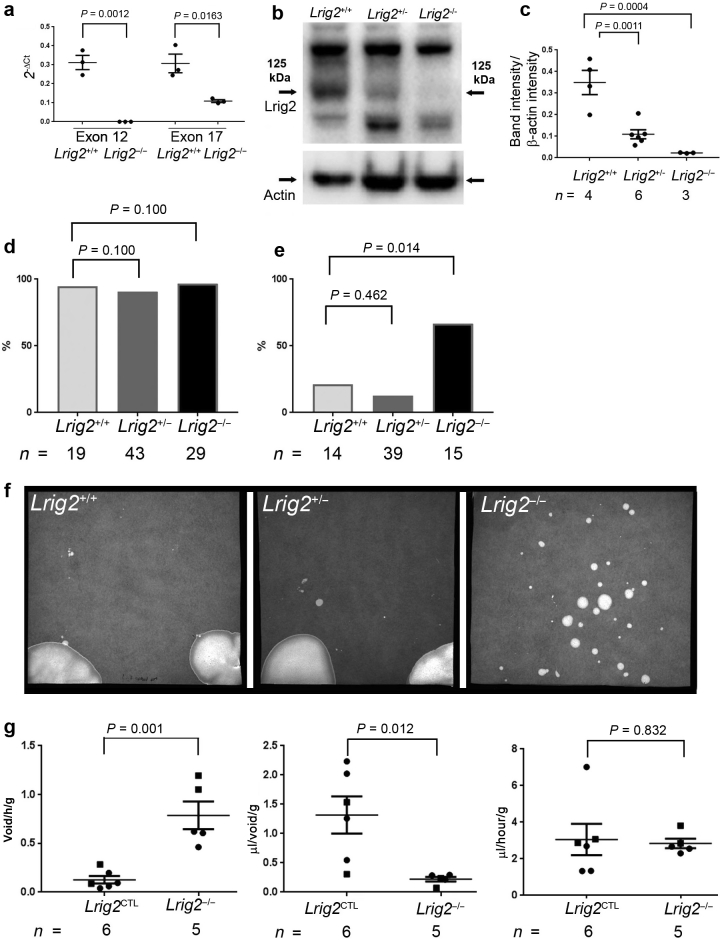
***Leucine*-*rich repeats and Ig-like domains 2****(****Lrig2****)***expression and urination patterns in mice.** (**a**) Quantitative reverse transcription–polymerase chain reaction demonstrated that *Lrig2* transcripts were present in wild-type bladders. In tissues from *Lrig2*^*–/–*^ littermates, transcripts were undetectable using exon 12 primers (*P* = 0.0012, 2-tailed unpaired Student *t*-test), and there were diminished levels of transcripts in *Lrig2*^*–/–*^ versus *Lrig2*^*+/+*^ tissues as assessed by exon 17 primers (*P* = 0.0163, 2-tailed unpaired Student *t*-test, *n* = 4). (**b**) Western blotting indicated that Lrig2, the 125-kDa band, was present in wild-type and heterozygous bladders but was not detected in homozygous mutant tissues. The panel below shows lysates from the same 3 organs probed for the housekeeping protein, β-actin (Actin). (**c**) Semiquantification of Western blot signals showed diminished values in both *Lrig2*^*+/–*^ (*P* = 0.0011) and *Lrig2*^*–/–*^ (*P* = 0.0004) tissues compared with wild types (ordinary one-way analysis of variance with Tukey’s multiple comparisons test). In (**a**) and (**c**), error bars are SEM. (**d**) Frequency of necropsy bladders containing urine in neonatal mice. Bladders from wild-type, heterozygous, and homozygous mice nearly always contained urine (*P* = 0.100, Fisher exact test). (**e**) Frequency of bladders containing urine at necropsy at 2 weeks after birth. *Lrig2*^*–/–*^ bladders contained urine significantly more often than did wild types (*P* = 0.014, Fisher exact test), whereas heterozygous bladders were similar to wild types (*P* = 0.462, Fisher exact test). (**f**) Urination patterns in weaned mice as assessed by voided stain on paper. The wild-type and heterozygous mice produced a few large voids in the observation period, whereas the *Lrig2*^*–/–*^ mouse produced many smaller voids in the same period. (**g**) Left panel: numbers of voids per unit time, with a significant increase in homozygous mutants versus control subjects (*P* = 0.001). Middle panel: volumes of urine per void, with a significant decrease in homozygous mutants versus control subjects (*P* = 0.012). Right panel: total volumes of urine produced per unit time, with no significant difference (*P* = 0.832) between control (comprising wild-type and heterozygous mice) and *Lrig2*^*–/–*^ littermates. Data are mean ± SEM, with values factored for weights of individual mice, with comparisons using 2-tailed unpaired Student *t*-test. Note that increased frequency of urination was observed in both male (boxes) and female (circles) homozygous mutant mice. To optimize viewing of this image, please see the online version of this article at www.kidney-international.org.

**Figure 2:**
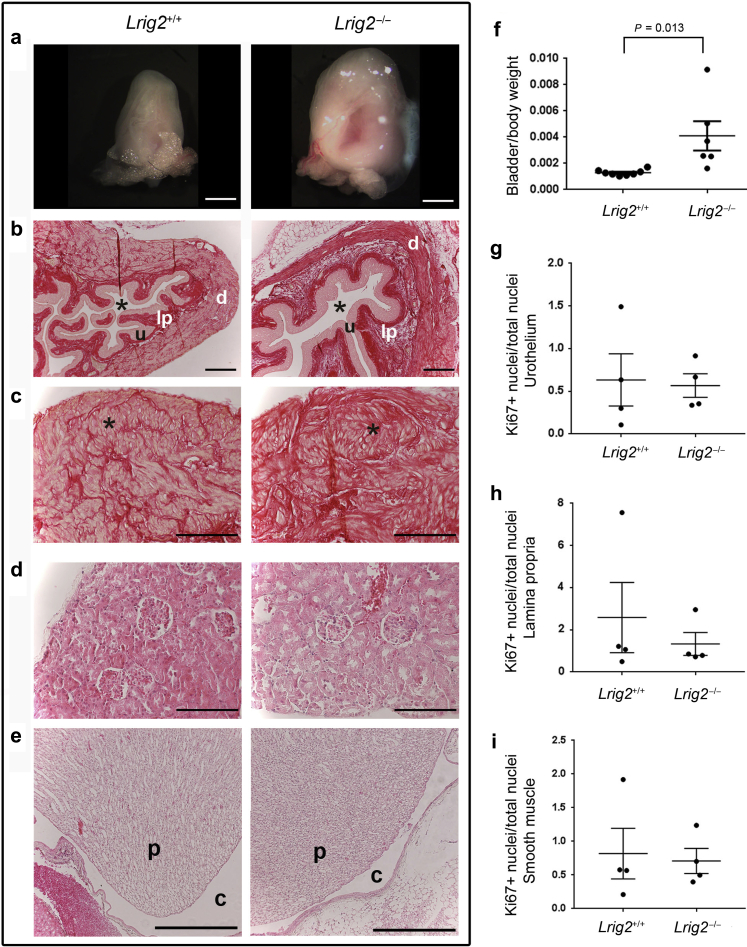
**Adult renal tract histology.** (**a**) Autopsy bladders: note the larger mutant organ. Bars = 1 mm. (**b**,**c**) Picrosirius red staining of bladder sections. Asterisk in (**b**) shows lumen. Note the more prominent lamina propria (lp in **b**) and detrusor smooth muscle (DSM) muscle bundles (* in **c**) in the mutant versus wild type. u, urothelium; d, DSM. (**d**,**e**) Picrosirius red staining of kidney sections showing similar appearances of the cortex (**d**) and deep medulla (**e**) in each genotype. p, papilla; c, calyx. Bars in (**b**–**e**) = 50 μm. (**f**) After draining urine from bladders, weights of mutant organs (*n* = 6, age between 2 and 12 months), shown here factored for body weight, were significantly (*P* = 0.013) greater than control subjects (*n* = 9, age 2–12 months). (**g**–**i**) Immunostaining adult bladders for Ki67 revealed no significant differences in percent proportions of positive nuclei in urothelial (**g**; *P* = 0.855), lamina propria (**h**; *P* = 0.500), and detrusor (**i**; *P* = 0.804) layers of *Lrig2*^*–/–*^ homozygous mutants (*n* = 4) and control subjects (*n* = 4). Ranges of total nuclei counted for each layer: urothelium, 2000–8000; lamina propria, 3500–10,000; and detrusor, 8000–20,000. Bars in (**f**–**i**) are means ± SEMs. To optimize viewing of this image, please see the online version of this article at www.kidney-international.org.

**Figure S1:**
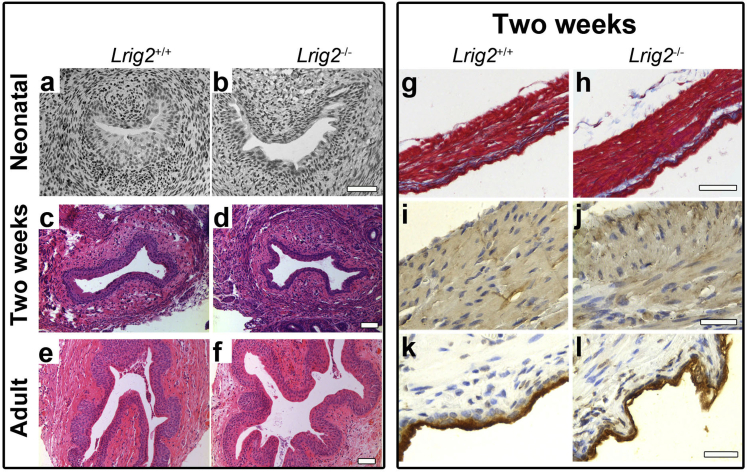
Neonatal and 2-week urinary bladder histology. (**A–F**) Bladder outflow: 5-μm paraffin sections cut transversely across the outflow comparing wild-type with *leucine-rich repeats and Ig-like domains 2* (*Lrig2*)^–/–^ mutants at neonatal, 2-week, and adult stages. All had patent outflow tracts. Bars = 100 μm. (**G–L**) Bladder body: 2-week bladder sections were reacted with Masson’s trichrome (**G** and **H**; muscle and urothelium = red and collagen = light blue), or muscle immunostained (brown) for α-smooth muscle actin (**I,J**), or urothelium immunostained (brown) for uroplakin II (**K,L**). Note that staining patterns were similar in *Lrig2*^–/–^ and *Lrig2*^*+/+*^ bladders. Bars = 50 μm.

**Figure S2:**
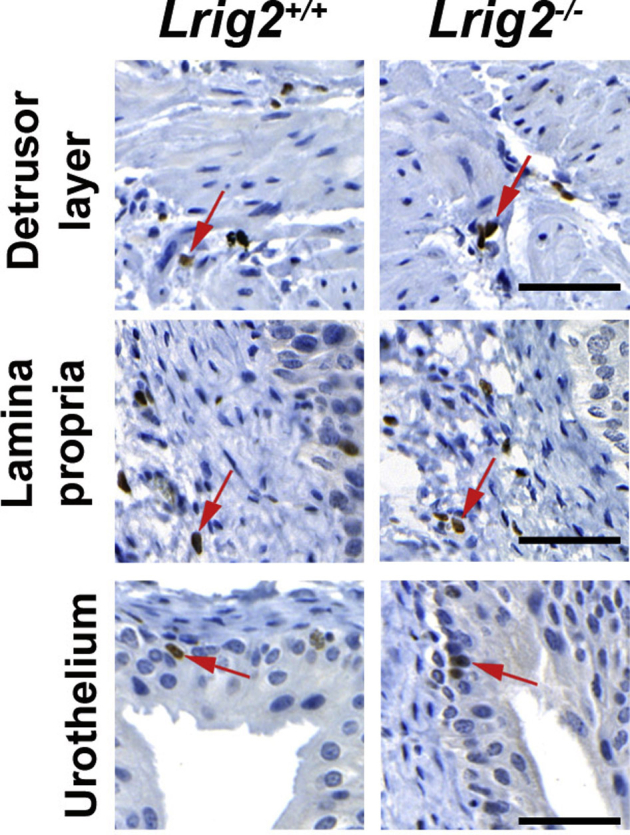
Ki67 immunostaining of adult bladders. All sections were counterstained with hematoxylin. Panels show high-power views of detrusor, lamina propria, and urothelial layers in wild types and mutants. Selections of nuclei positive for Ki67 are indicated by arrows. Bars are 50 = μm.

We used mice with a targeted deletion of *Lrig2* exon 12,^19^ which introduces a frameshift preceding the region encoding the transmembrane domain, generating multiple stop codons. Mice had been maintained on a C57BL6 background for more than 10 generations, and mating heterozygotes generated offspring for study. Upon analyzing the bladder and outflow tract unit, quantitative reverse transcription–polymerase chain reaction (qRT-PCR) detected *Lrig2* in wild-type neonatal (first postnatal day) samples. In *Lrig2*^–/–^ littermates, transcripts were undetectable using exon 12 primers and were significantly diminished (*P* = 0.0163) to one third of wild-type levels using exon 17 primers (Figure 1a). Using an antibody to a cytosolic Lrig2 epitope absent in Lrig1 and Lrig3,^19^ Lrig2 was detected as a 125-kDa band in Western blots of wild-type bladders (Figure 1b). Lrig2 was absent in *Lrig2*^–/–^ littermates, with intermediate levels in *Lrig2*^+/–^ samples (Figure 1b and c). Neonatal mice of the 3 genotypes had similar weights. At 2 weeks, *Lrig2*^–/–^ mice had gained less weight than the other genotypes, being on average 14% lighter than *Lrig2*^*+/+*^ or *Lrig2*^+/–^ littermates. Neonatal necropsy bladders, examined within 5 minutes of cervical dislocation, were classified as empty or containing urine by visual inspection. Approximately 90% of bladders contained urine in all 3 genotypes (Figure 1d). At 2 weeks (Figure 1e), 67% of *Lrig2*^–/–^ mouse bladders contained urine, a significant (*P* = 0.014) increase versus 21% of wild-type bladders. The frequency of heterozygous bladders containing urine was similar to wild types. These and adult mice were studied by the voided stain on paper (VSOP) technique^20^ where they urinate onto filter paper, with numbers and sizes of spots measured. Wild-type and heterozygous *Lrig2* mice voided once or twice per hour, whereas homozygous *Lrig2* mutant mice voided up to 20 times per hour, producing smaller spots than *Lrig2*^*+/+*^ or *Lrig2*^*+/*–^ mice (Figure 1f). Quantification (Figure 1g) showed that *Lrig2*^–/–^ mice voided more frequently (*P* = 0.001), and their average volume per void was lower (*P* = 0.0124) compared with control subjects. Total urine voided per time was similar in control (*Lrig2*^*+/+*^ and *Lrig2*^*+/–*^) mice and *Lrig2*^–/–^ mice. These aberrant urination patterns, observed from 14 days to adulthood, resemble those in UFS.^5^, ^6^ UFS uropathy is not sex limited,^5^ and both male and female *Lrig2*^–/–^ mice had abnormal urination. Ink was injected into the bladder lumen at autopsy, and upon gentle palpation, ink exited the urethra. Upon histologic examination (Supplementary Figure S1A–F), bladder outflows were patent in neonatal, juvenile, and adult *Lrig2*^–/–^ mice. Histology of the bladder body was similar in wild types and mutants at 2 weeks (Supplementary Figure S1G–L). Adult *Lrig2*^–/–^ bladders appeared larger than wild types (Figure 2a). Histology with picrosirius red, a collagen reactive dye, revealed prominent staining in mutant lamina propria and detrusor (Figure 2b and c). *Lrig2*^–/–^ kidneys showed no scarring (Figure 2d and e). After draining urine from bladders, mutant organs were significantly heavier than those of control subjects (Figure 2f). Immunostaining adult bladders for Ki67 (Supplementary Figure S2) revealed similar proportions of proliferating nuclei in mutants and control subjects (Figure 2g–i). Detrusor nuclei number was similar (*P* = 0.36) in control mice (mean ± SEM, 431 ± 88/mm^2^, *n* = 4) and mutants (453 ± 53/mm^2^, *n* = 4).

### Transcriptome analyses of *Lrig2* mutants

**Figure 3:**
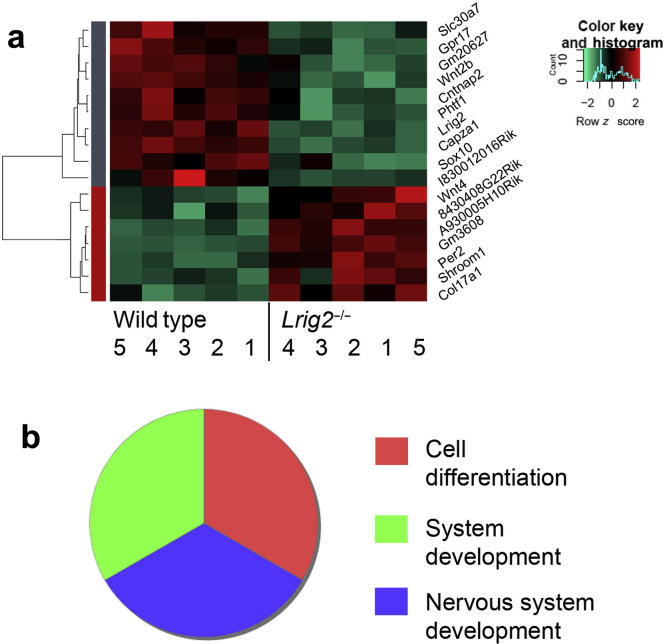
**RNA sequencing analyses.** (**a**) Unsupervised hierarchical clustering distinguished between wild-type (*n* = 5) and homozygous *Leucine-rich repeats and Ig-like domains 2* (*Lrig2*)^*–/–*^ (*n* = 5) bladders and their attached outflow tracts. Rows are expression levels denoted as the *z* score, displayed in a high–low (red–green) color scale. (**b**) Gene ontology analysis using the Panther Classification System. The only tissue-specific enriched term was *nervous system development*.

To investigate the molecular milieu before urination defects became established, we undertook RNA sequencing of neonatal bladders and their attached outflow tracts harvested from 5 *Lrig2*^*+/+*^ *and Lrig2*^–/–^ littermate pairs. We used females because the male tract includes the prostatic rudiment, with a transcriptome that could complicate interpretation of resulting arrays. The data set is available from *ArrayExpress* (E-MTAB-6089). Unsupervised hierarchical clustering distinguished between homozygous *Lrig2* mutants and wild types (Figure 3a). Thirteen significantly differentially regulated known transcripts were identified after adjusting for multiple comparisons. As expected, *Lrig2* was one of the downregulated transcripts. Gene ontology analysis (Figure 3b) revealed enrichment of cell differentiation and system development terms. The only tissue-specific enriched term was nervous system development. Several of these transcripts encode proteins implicated in neurobiology, as detailed in the Discussion section.

### UFS proteins in bladder nerves

**Figure 4:**
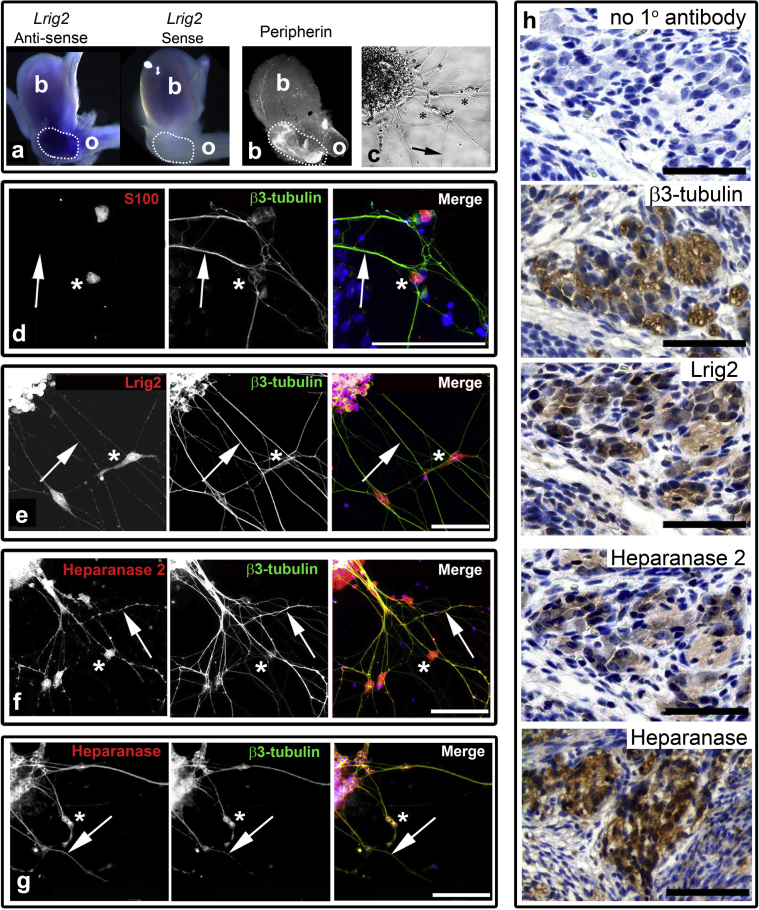
**Neuronal localization of leucine-rich repeats and Ig-like domains 2 (Lrig2) and heparanase 2.** (**a**) Whole-mount *in situ* hybridization for *Lrig2*. Side views of embryonic day 15 wild-type bladders, with the left frame showing anti-sense probe and the right frame showing control sense probe. Note the strong signal (blue) at the junction of the body (*b*) and outflow (*o*) corresponding to the location of the pelvic ganglia (dotted outline). Blue color in the body is nonspecific trapping in the lumen in both genotypes. (**b**) Similar side view of whole-mount embryonic bladder immunostained for peripherin (white). The pelvic ganglion (dotted outline) is strongly positive. *b*, body; *o*, outflow. (**c**) Phase contrast of embryonic pelvic ganglion explant. After 1 day in culture, processes (arrow) had emerged from the cell mass (upper left of the frame), as had cell bodies (asterisks). (**d**–**g**) Immunocytochemistry of pelvic ganglia explants. The first 2 frames in each row show signals (white) for individual proteins (red or green) named in each frame. The final (merge) frame in each row shows double immunostaining, with nuclei stained with 4′,6-diamidino-2-phenylindole (blue). (**d**) The glial marker, S100, was immunodetected in cell bodies (asterisks) that had emerged from the explanted mass, whereas emerged processes (arrows) immunostained for the neuronal microtubule protein β3-tubulin. (**e**) Emerged cell bodies (asterisks) and processes (arrows) immunostained for Lrig2. (**f**) Emerged cell bodies (asterisks) and neuronal processes (arrows) immunostained for heparanase 2. (**g**) Neuronal processes (arrows) immunostained for heparanase, the classical heparanase. Asterisks indicate the cell body. Bars in (**d**–**g**) = 100 μm. (**h**) Bright field immunohistochemistry of neonatal pelvic ganglion immunostained (brown) for β3-tubulin, Lrig2, heparanase 2, and the classical heparanase, with nuclei stained with hematoxylin. Note that all 3 antibodies label large cells: these are the cell bodies of autonomic neurons. Bar = 50 μm. To optimize viewing of this image, please see the online version of this article at www.kidney-international.org.

**Figure S3:**
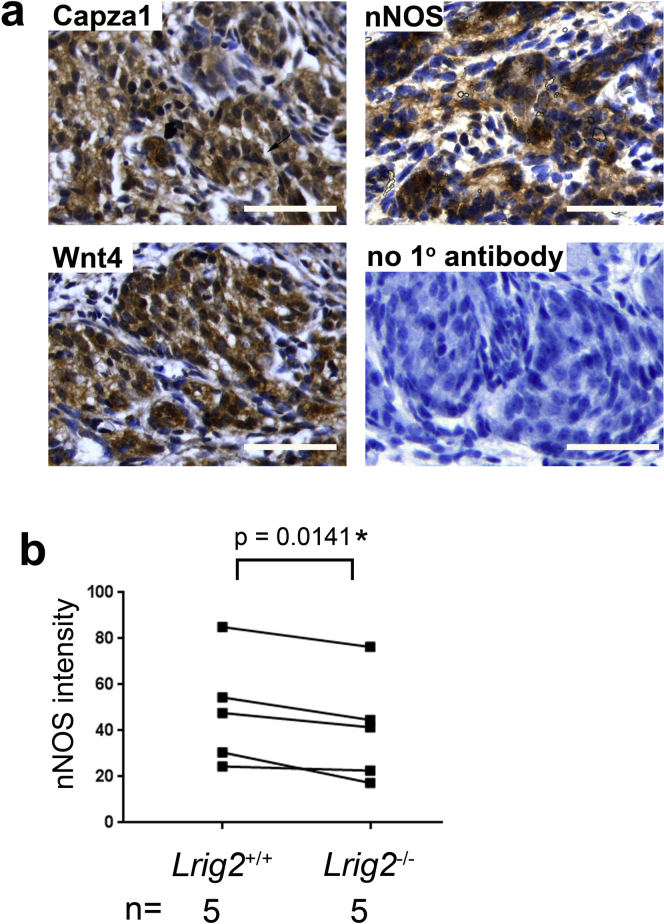
Immunostaining of neonatal pelvic ganglia. (**A**) Immunohistochemistry for Wnt family member 4 (Wnt4) and capping actin protein of muscle Z-line subunit alpha 1 (Capza1), encoded by 2 transcripts highlighted in unsupervised hierarchical clustering (Figure 3) and neuronal nitric oxide synthase (nNOS), showed that all 3 were detectable in wild-type neonatal pelvic ganglia. The bottom left frame is a negative control with no primary antibody. All sections were counterstained with hematoxylin, with positive immunohistochemical signal in brown. (**B**) Quantification of nNOS immunohistochemical signals in neonatal ganglia showed a small but statistically significant reduction (Student *t*- test, *P* = 0.014) in *leucine-rich repeats and Ig-like domains 2 (Lrig2)*^*–/–*^ tissues (mean ± SEM in 5 control subjects = 48.4 ± 10.7 arbitrary units and in 5 mutants = 40.5 ± 10.4). nNOS immunostaining was semi-quantified by taking the average pixel intensity reading for the pelvic ganglia region only in ImageJ.

The RNA sequencing results were generally consistent with the hypothesis that the UFS bladder has a neural component, and this led us to examine nerve patterns in *Lrig2*^–/–^ bladders. In mice, the 2 pelvic ganglia flank the bladder outflow.^3^, ^21^ We studied wild-type mice two thirds through gestation, when these ganglia are composed of loose aggregates^10^ of neural crest-derived cells^22^ and the bladder rudiment has acquired well-defined body and outflow tract. *Lrig2 in situ* hybridization demonstrated prominent signals at the junction of the body and outflow tract (Figure 4a), the sites of pelvic ganglia immunostaining for peripherin, a neural intermediate filament (Figure 4b). Embryonic pelvic ganglia were explanted and cultured, adapting published protocols.^23^, ^24^ During the next day, processes and cell bodies emanated from cell aggregates (Figure 4c). The processes immunostained for the neuronal microtubule protein β3-tubulin (Figure 4d) and migrating cell bodies immunostained for S100B, a calcium-binding protein expressed by autonomic ganglia glia.^25^ Lrig2 was immunodetected in cell bodies within explanted aggregates; it was immunodetected faintly in the emerged neurites and more prominently in emerged glia-like cells (Figure 4e). Heparanase 2 was immunodetected in cell bodies, neurites, and glia-like cells (Figure 4f). Heparanase, the classical heparanase,^11^ was immunodetected in neurite-like structures (Figure 4g). Upon histologic examination, pelvic ganglia of neonatal wild-type mice immunostained for Lrig2, heparanase 2, and heparanase (Figure 4h). Immunohistochemistry for Wnt4 and Capza1, encoded by 2 transcripts highlighted in unsupervised hierarchical clustering (Figure 3), showed that they were detectable in neonatal pelvic ganglia (Supplementary Figure S3). In wild-type neonatal ganglia, 9% of nuclei were Ki67^+^. Pelvic ganglia in *Lrig2*^–/–^ littermates had a similar (*P* = 0.190) proportion of positive nuclei (*n* = 4 mice, each genotype).

### Aberrant patterns of nerves in bladders of mice mutant for *Lrig2* or *Hpse2*

**Figure 5:**
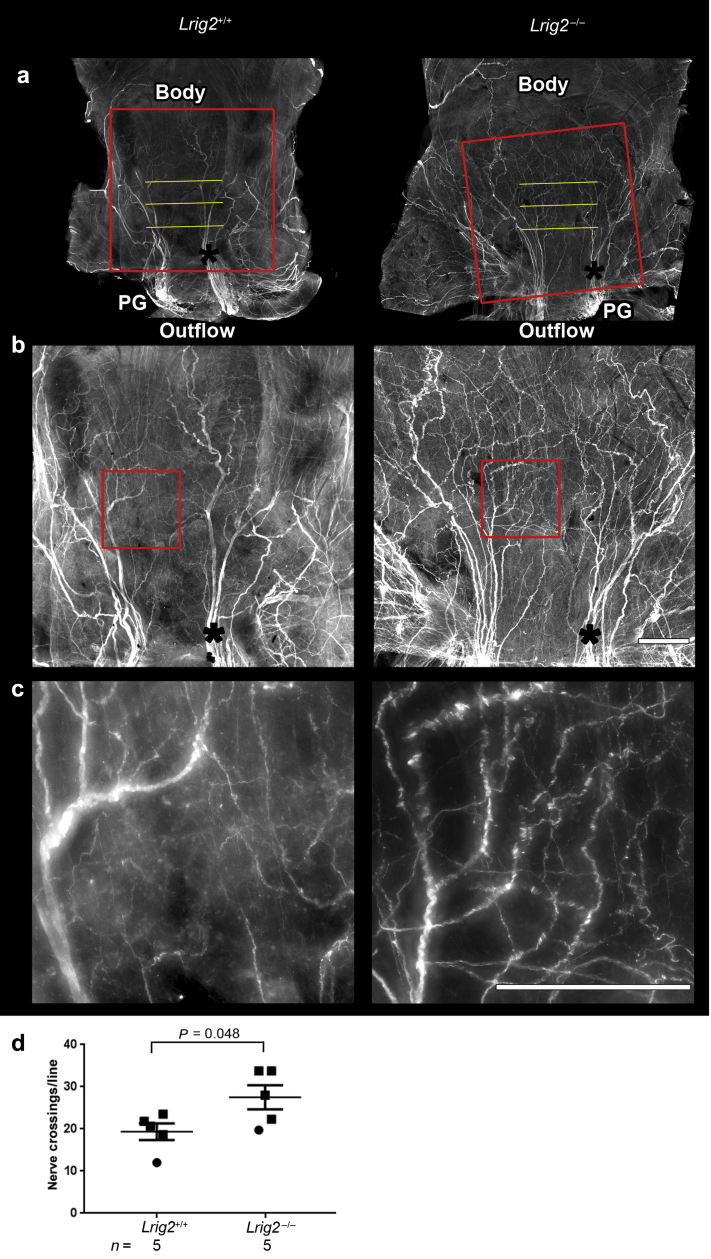
**Aberrant nerves in the *leucine-rich repeats and Ig-like domains 2* (*Lrig2*)**^***–/–***^**bladder body.** (**a**) Whole wild-type (left column) and *Lrig2*^*–/–*^ (right column) bladder sheets immunostained for the neural protein peripherin. The top (cranial) part of the bladder is uppermost. Yellow lines denote regions in the bladder body where peripherin+ nerve crossings were quantified. Red boxes indicate regions magnified in the higher-power images, directly below. Black asterisks indicate the large bundles of nerves in the bladder body that connect with the pelvic ganglia (*PG*). (**b**,**c**) Higher magnifications showing large nerve bundles (asterisks) and finer fascicles in the bodies of the organs. Note the apparently more prominent peripherin+ structures in the *Lrig2*^*–/–*^ versus the wild-type littermate bladder. Bars = 200 μm. (**d**) Quantification of peripherin+ structures (i.e., nerves crossing the yellow grid lines) showed a significant increase (average 40%) of nerves in *Lrig2*^*–/–*^ versus *Lrig2*^*+/+*^ bladders (mean ± SEM, *P* = 0.048, 2-tailed unpaired Student *t*-test, *n* = 5). Male bladders are indicated by squares and female bladders by circles. To optimize viewing of this image, please see the online version of this article at www.kidney-international.org.

**Figure 6:**
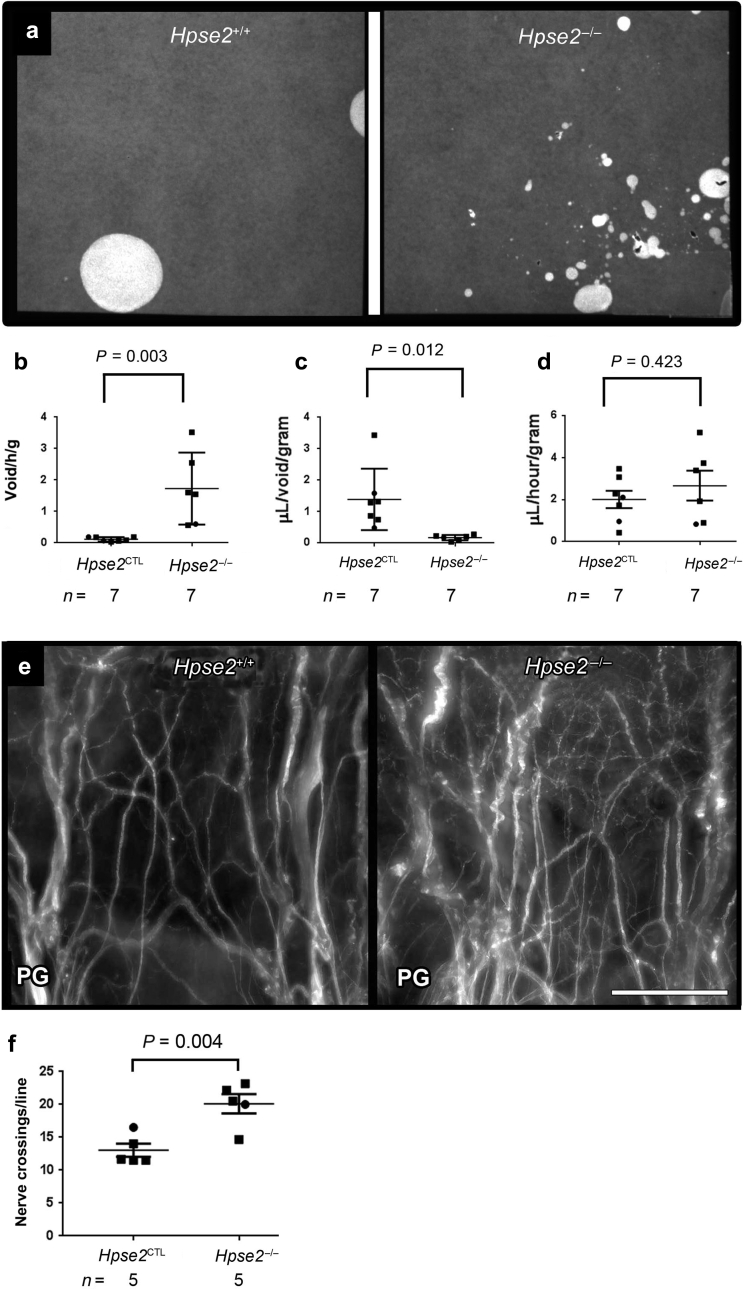
**Urination defects and aberrant nerves in the bladder of *heparanase 2* (*Hpse2*) mutant mice.** (**a**) Voided stain on paper urination patterns produced by an *Hpse2*^*–/–*^ mouse (right frame) and a wild-type littermate (left frame). (**b**) Numbers of voids per unit time, with a significant (*P* = 0.003) increase in homozygous mutants versus control subjects. (**c**) Volumes of urine per void, with a significant (*P* = 0.012) decrease in homozygous mutants versus control subjects. (**d**) Total volumes of urine produced per unit time, with no significant (*P* = 0.423) difference between control (comprising wild-type and heterozygous mice) and *Hpse2*^*–/–*^ littermate pairs. Values in (**b**–**d**) are mean ± SEM and are factored for weights of individual mice. Comparisons were made using 2-tailed unpaired Student *t*-tests. (**e**) Whole-mount immunostaining of 1-week postnatal bladders. Note increased peripherin+ (white) structures in the homozygous *Hpse2* mutant (right frame) versus the wild-type littermate (left frame) bladder. Bar = 200 μm. (**f**) Quantification of peripherin+ structures (i.e., nerves crossing grid lines, as for Figure 5a) confirmed a significant (*P* = 0.004) increase in *Hpse2*^*–/–*^ versus control bladders. Data are mean ± SEM, 2-tailed unpaired Student *t*-test, *n* = 5. Bars are 200 μm. To optimize viewing of this image, please see the online version of this article at www.kidney-international.org.

Bladders harvested from mice in the second week after birth were cut open, generating butterfly shaped sheets, and whole-mount immunostained for peripherin. In *Lrig2* mutant and wild-type littermate bladders, pelvic ganglia expressed peripherin, as did nerves at the junction of the outflow and the bladder base and nerves in the body (Figure 5). Representative bladders are shown in increasing magnifications in Figure 5a, b, and c. We noted 2 characteristic changes in mutants versus wild types. First, large nerve bundles in the bladder base (black asterisks, Figure 5a) continuous with the pelvic ganglia appeared to be less compactly bundled. Second, there appeared to be an increased density of smaller nerves in the bladder body (Figure 5b and c). Upon quantification by observers blinded to genotypes, a significant (*P* = 0.048) increase of peripherin+ structures was confirmed in *Lrig2* mutant bladders (Figure 5d). Using VSOP analyses (Figure 6a), *Hpse2* homozygous gene-trap mice^10^ produced significantly more voids per unit time (*P* = 0.003) than did control (wild-type and heterozygous) littermates (Figure 6b). Mutant void volumes were significantly (*P* = 0.012) smaller than those of control mice (Figure 6c), but with no significant difference (*P* = 0.423) in total volumes urinated per unit time (Figure 6d). These aberrant urination patterns resemble those reported in another *Hpse2* gene trap mouse.^12^ Bladder sheet immunostaining demonstrated bundles of peripherin+ nerves in bladder body that appeared more prominent in *Hpse2* mutants than in control bladders (Figure 6e). Quantification showed an increase (*P* = 0.004) of peripherin+ structures in *Hpse2*^–/–^ versus *Hpse2*^*+/+*^ littermates (Figure 6f). *Hpse2* and *Lrig2* lines were interbred, generating transheterozygotes—that is, mice carrying one mutant allele at each locus. Upon being studied by VSOP (*n* = 6 adults, a cohort containing males and females), their urination patterns appeared similar to wild-type or single heterozygous littermates.

**Figure 7:**
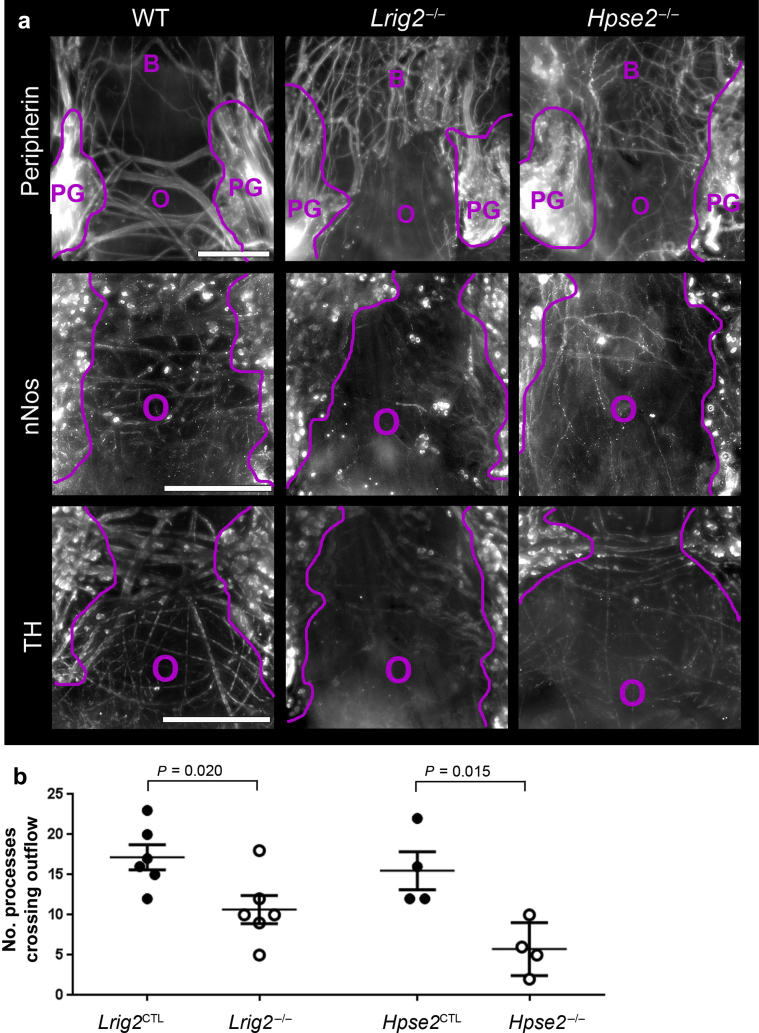
**Nerves around the bladder outflow tract.** (**a**) Whole-mount immunostaining of bladders in the second week after birth. Bladders were reacted with antibodies to peripherin, a pan-neuronal marker (top row); neuronal nitric oxide synthase (nNOS), a marker of nitrergic parasympathetic neurons (middle row); or tyrosine hydroxylase (TH), a sympathetic neuron marker (bottom row). Purple lines define borders of pelvic ganglia (*PG*), and the bladder body is denoted by *B* and the outflow by *O*. Neurons spanning the 2 ganglia were detected in wild-type (WT) bladders (first column) with all 3 markers. Nerves in this zone were less prominent in both *Lrig2* (middle column) and *Hpse2* (right column) mutant outflow tracts. In contrast, in both the *Lrig2* and the *Hpse2* mutant bladders depicted, peripherin+ nerves in the bladder body were denser in each mutant than in the WT organ. Bars = 200 μm. For peripherin immunostaining, 3 WT, 5 *Lrig2*^*–/–*^, and 5 *Hpse2*^*–/–*^ mice were studied. For nNOS immunostaining, 3 WT, 4 *Lrig2*^*–/–*^, and 5 *Hpse2*^*–/–*^ mice were studied. For TH immunostaining, 3 WT, 4 *Lrig2*^*–/–*^, and 5 *Hpse2*^*–/–*^ mice were studied. (**b**) Quantification of peripherin-positive nerves around the outflow tracts. Note the significant reductions in both *Lrig2* and also *Hpse2* homozygous mutants compared with control mice. To optimize viewing of this image, please see the online version of this article at www.kidney-international.org.

**Figure S4:**
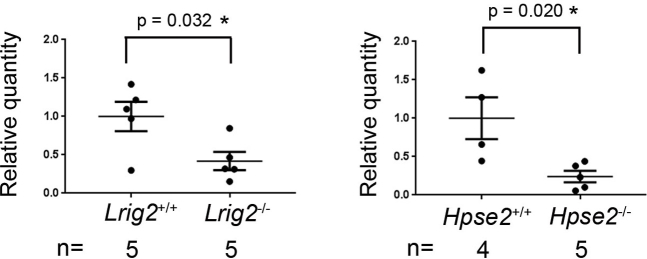
Quantitative reverse transcription–polymerase chain reaction for *nitric oxide synthase 1* (*Nos1*) in neonatal bladders and attached outflow tracts. For both *leucine-rich repeats and Ig-like domains 2 (Lrig2)* homozygous mutant tissues and the *heparanase 2* (*Hpse2)* mutant tissues, *Nos1* transcripts levels were reduced compared with littermate control subjects. Values are shown as mean ± SEM, with the value for the mean of each wild-type group defined as “1.”

**Figure S5:**
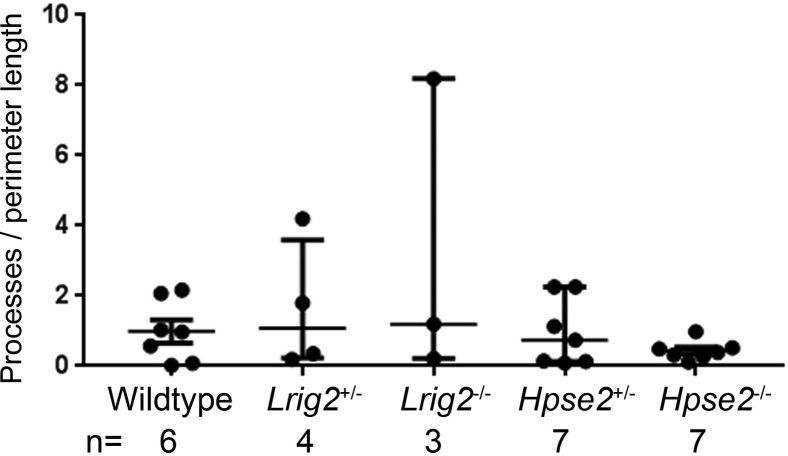
Quantification of processes emanated from pelvic ganglia explants. Fragments of embryonic pelvic ganglia were explanted and the numbers of processes that had emanated from them were counted after 1 day in culture. Results are shown as median and ranges, with values factored for the length of the perimeter in each explant. The wild-type median was defined as “1.” There were no significant differences (all *P* > 0.05) between wild-type explants and either *leucine-rich repeats and Ig-like domains 2* (*Lrig2*) or *heparanase 2* (*Hpse2*) mutants as assessed by Kruskal-Wallis tests. The number of ganglia assessed are shown in the figure.

Nerves around the outflow tract were analyzed in the second week after birth (Figure 7a). In wild types, peripherin immunostaining revealed ganglia flanking the outflow tract, with prominent nerve bundles between them, lying in the dorsal side of the outflow. In *Lrig2* or *Hpse2* homozygous mutants, a spectrum of aberrations was observed, from disorganized to almost absent peripherin+ nerves. Next, we used markers of specific neural subtypes: anti-nNOS immunostaining to detect nitrergic parasympathetic nerves and anti-tyrosine hydroxylase immunostaining to mark sympathetic nerves. In control subjects, a subset of cell bodies in pelvic ganglia were nNOS^+^, and fine bundles connecting the pelvic ganglia were also nNOS^+^. nNOS^+^ cell bodies also were observed in ganglia of *Lrig2*^–/–^ or *Hpse2*^–/–^ bladders. In contrast, patterns of nNOS^+^ connecting nerves appeared aberrant in either mutant, sometimes with barely any positive processes detected. In wild-type outflow tracts, anti-tyrosine hydroxylase immunostaining was detected in a subset of cell bodies in ganglia and in nerve bundles spanning pelvic ganglia. In mutant outflow tracts, anti-tyrosine hydroxylase immunostaining+ bundles appeared disorganized or reduced. Numbers of peripherin-positive nerves around outflow tracts (Figure 7b) were significantly less in either *Lrig2* or *Hpse2* homozygous mutants compared with control mice. In histologic sections of neonatal bladders, nNOS was immunodetected in pelvic ganglia (Supplementary Figure S3), with a small but statistically significant reduction of signal intensity in *Lrig2*^–/–^ ganglia. qRT-PCR for *Nos1* in neonatal bladders and outflow tracts showed significantly reduced levels in *Lrig2* and *Hpse2* mutants (Supplementary Figure S4). This finding was consistent with the RNA sequencing, which revealed that *Lrig*^–/–^ mutant bladders had average *Nos1* levels of 48% of wild-type values (*P* = 0.001*,* when multiple comparison testing was not used). Numbers of processes emanated from explanted pelvic ganglia fragments were similar in mutants and control subjects (Supplementary Figure S5).

### *LRIG2* variants in nonsyndromic bladder outlet obstruction

**Figure 8:**
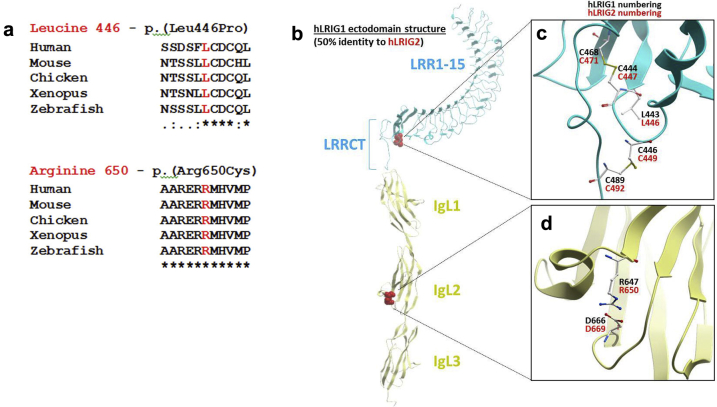
***Leucine-rich repeats and Ig-like domains 2* (*LRIG2*) missense variants.** (**a**) Conservation of Leu446 and Arg650 between human, mouse, chicken, xenopus, and zebrafish protein sequences. An asterisk indicates positions which have a single, fully conserved residue; a colon indicates conservation between groups of strongly similar properties, scoring >0.5 in the Gonnet point accepted mutation 250 matrix; a period/full stop indicates conservation between groups of weakly similar properties, scoring <0.5 in the Gonnet point accepted mutation 250 matrix. (**b**) Extracellular domains of human LRIG1 (*hLRIG1*) that has approximately 50% sequence similarly to human LRIG2. Leucine-rich repeats (*1–15*) and Ig-like domains (*1–3*) are depicted. (**c,d**) These 2 frames show details of the regions containing the Leu446Pro (*L446*) and the Arg650Cys (*R650*) LRIG2 missense variants, respectively.

Putative loss-of-function *LRIG2* variants have been described in UFS.^13^ We also reported compound heterozygous missense *LRIG2* variants (p.Arg550Cys in exon 13 and p.Ile852Phe in exon 16) in a patient with a non-neurogenic neurogenic bladder but with a normal smile.^13^ To seek *LRIG2* variants in other uropathies, we used Sanger sequencing for *LRIG2* in children with chronic kidney failure from the 4C Study (http://www.4c-study.org/). We focused on a subset of 155 patients with urinary tract symptoms (e.g., incontinence of urine and poor stream) and/or signs (e.g., abnormal urinary tract radiology). Two individuals were found to have homozygous missense variants in *LRIG2*. One harbored c.1337T>C; p.(Leu446Pro) in exon 12, absent in control databases including >120,000 persons in the Genome Aggregation Database (http://gnomad.broadinstitute.org) and predicted to be deleterious by *in silico* tools including Sorting Intolerant From Tolerant, Polymorphism Phenotyping version 2), and MutationTaster. This individual had a non-neurogenic neurogenic bladder but lacked UFS facial features. Another patient harbored c.1948C>T; p.(Arg650Cys) in exon 14 of *LRIG2*. This characteristic has been reported in 8 of 277158 alleles in the Genome Aggregation Database but never in the homozygous state and is predicted to be deleterious by Sorting Intolerant From Tolerant, Polymorphism Phenotyping version 2, and MutationTaster. The patient had congenital bladder outlet obstruction but lacked UFS facial features. It was not possible to obtain to access relatives. The 2 cases were Turkish, so we screened 85 healthy Turkish control subjects but failed to find either Leu446Pro or Arg650Cys variants in 170 chromosomes. Figure 8a shows that both Leu446 and Arg650 are conserved between human, mouse, chicken, xenopus, and zebrafish protein sequences. As detailed in the Supplementary Results text and depicted in Figure 8b–d, these missense *LRIG2* variants may have an impact on the structural integrity of the LRIG2 extracellular region by affecting disulphide bonds for Leu446Pro and a salt bridge for Arg650Cys. *LRIG2* exons 12, 13, 14, and 16*,* known to harbor missense variants in non-neurogenic neurogenic bladder, were sequenced in 192 index cases from the United Kingdom Vesicoureteric Reflux (VUR) DNA Collection.^26^ They had familial primary nonsyndromic VUR, ureter malformations without anatomic bladder outlet obstruction, non-neurogenic neurogenic bladder, or extrarenal malformations. Five rare heterozygous *LRIG2* variants were identified in index cases, but no biallelic variants were detected. Only 2 variants segregated with disease and neither was uniformly predicted.

## Discussion

In rats, neuromuscular functional maturation of detrusor smooth muscle occurs in the few first weeks after birth.^27^ Our results show that bladders in newborn mice, whether wild type or *Lrig2* mutant, are nearly always full at necropsy. In contrast, by 2 postnatal weeks, wild-type and *Lrig2* heterozygous necropsy bladders are usually empty, whereas bladders of *Lrig2*^*–/–*^ mice usually contain urine. Therefore, a bladder emptying mechanism is becoming established in wild-type and heterozygous mice between birth and 2 weeks of age, but this is defective in *Lrig2*^–/–^ mice. In older mice, *Lrig2*^–/–^ mutants urinated more frequently than did wild types but their individual voids were of lower volume. These dysfunctional patterns resemble those in people with UFS.^5^ *Lrig2*^–/–^ mouse urination defects were not caused by anatomic blockage of the outflow tract, consistent with functional bladder outflow obstruction. Urination patterns of *Lrig2* heterozygous mice appeared normal. Again, this finding is similar to that in human families affected by UFS because members who carry only one variant *LRIG2* allele are phenotypically normal.^13^ The fact that *Lrig2* heterozygous mice had normal urination despite containing markedly less Lrig2 protein compared with wild-type bladders suggests that future treatments for UFS—for example, using gene therapy^28^—may be effective if they produce only a modest amount of Lrig2 protein. A previous report stated that another *Lrig2* homozygous mutant line^29^ had normal bladder pressure as assessed by cystometrography.^12^ This mouse was created by random insertion of a gene trap into exon 11, resulting in fused *Lrig2* and β-galactosidase and neomycin resistance (*βgeo*), with a low level of Lrig2 detectable in homozygous tissues.^29^ Perhaps the different mode of targeting *Lrig2*, or background strain differences, account for the apparently discrepant bladder findings between this and the current study.

**Figure 9:**
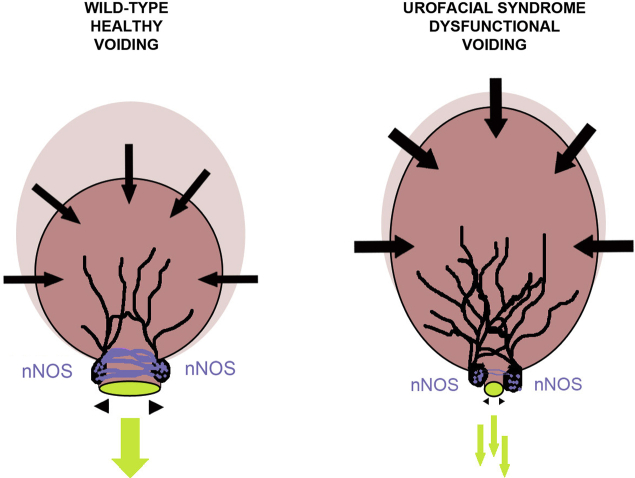
**Dysfunctional voiding in urofacial syndrome (UFS).** In the healthy bladder (left) a robust urinary stream and efficient emptying are facilitated by full dilatation of the outflow tract–driven neuronal nitric oxide synthase (nNOS) nerves. In the UFS bladder (right) the stream is poor because the outflow fails to fully dilate, which is associated with downregulated nNOS. The detrusor muscle in the body of the UFS bladder is overactive, which is associated with an abundance of nerves in the body of the organ. Contractile forces in the bladder body are shown by black arrows facing inward, and outlet dilatation is shown by black arrowheads facing outward. Nitrergic (nNOS) autonomic nerves around the outflow are depicted in purple, and those in the body (presumed cholinergic) are depicted in black. Urine is depicted in light green. The body of the uncontracted bladder is represented in lilac, and the voiding bladder body is shown in pink.

Mice homozygous for either *Lrig2* or *Hpse2* mutations had increased nerve density within the bladder body and decreased density around the outflow tract. LRIG2, heparanase 2, and heparanase itself were present in neural cells emanating from explanted embryonic pelvic ganglia and are present in intact pelvic ganglia. Our observations provide strong evidence that a peripheral neuropathy is part of the pathobiology of UFS bladder disease, whether caused by *HPSE2* or *LRIG2* variants. Figure 9 summarizes aberrations in the UFS bladder, linking established clinical observations with the new data.

Considering the UFS pathology, Lrig2 and heparanase 2 also may be implicated peripherally in the facial nerve biology because persons with UFS have a characteristic grimace upon smiling.^5^, ^6^ It had been hypothesized that a lesion affecting the pontine micturition center and the facial nerve nuclei, which control urination and facial expression, respectively, could account for the disparate phenotypes observed in the disease.^5^ However, the fact that the micturition center and the facial nerve nucleus are discrete structures makes a single lesion theory unlikely.^30^ Further studies may resolve whether molecules mutated in UFS also play roles in the facial nerve and brainstem.

Lrig2 is implicated in vestibular and auditory nerve function,^29^ and it modulates optic nerve regeneration.^31^ Another member of the Lrig family, Lrig1, acts as a break on primary dendrite formation and dendritic branching of hippocampal neurons.^32^ Heparanase, the enzyme inhibited by heparanase 2,^11^ modulates neurite outgrowth from pheochromocytoma cells,^33^ and heparanase protects against axonal degeneration after sciatic nerve injury.^34^ Moreover, embryonic frogs depleted of heparanase 2 have disorganized peripheral nerves.^35^ The current data showing aberrant nerves in *Hpse2 or Lrig2* mutant bladders therefore add to a growing body of literature that the heparanase 2 and Lrig axis modulates neural biology. A biological explanation for the abnormal patterns of nerves in mutant and wild types is not yet clear. Our current study, however, detected no increased proliferation in cells within neonatal pelvic ganglia of *Lrig2* homozygous mutant mice versus wild types. We also found no significant differences in outgrowth of processes from explanted pelvic ganglia, comparing either *Hpse2* or *Lrig2* tissues with wild-type explants. One caveat is that it is not possible to know which neurites would have been destined to populate the bladder body or the outflow. The same applies to the nature of individual neuronal cell bodies visualized in pelvic ganglia histology. These considerations could be important because we counted more peripherin-positive nerves in the bladder body but fewer around the outlet. Bladders from adult *Lrig*^*–/–*^ mutants were bulkier than those of control subjects, with prominent picrosirius red staining upon histologic examination. Indeed, prominent lamina propria collagen was reported in *Hpse2* mutant bladders in their third postnatal week.^12^ These changes may be secondary to bladder outlet obstruction.

Humans who have UFS associated with mutations of either *HPSE2* or *LRIG2* have similar bladder phenotypes,^6^ which is consistent with the hypotheses that the proteins encoded by these 2 genes maintain neural health by independent pathways or that the proteins interact, directly or indirectly, during their normal functions. Transheterozygote mice carrying one mutant allele at both the *Lrig2* and *Hpse2* loci did not have abnormal urination patterns, perhaps arguing against the idea that the encoded proteins directly interact. However, this observation also may be explained by a lack of haploinsufficient effects. It also should be noted that heparanase 2 may play biological roles in certain diseases unrelated to UFS, for example, in Alzheimer’s disease^36^ and head and neck cancer growth.^37^ The same applies to Lrig2 that has been implicated in tumor biology.^15^, ^16^, ^19^

nNOS is a well-established mediator of smooth muscle relaxation, including in the lower urinary tract.^38^ Indeed, *Nos1* homozygous mutant mice have incomplete bladder emptying caused by defective neurogenic relaxation of the outflow tract.^4^ We observed that the outflow tracts of mutant *Lrig2* or *Hpse2* bladders had a reduced number of nitrergic nerves compared with control subjects; if this finding translates to humans, the mouse observations could explain why the UFS bladder outflow fails to dilate, a key defect in the syndrome.^5^ We also documented an increased density of nerves in the bodies of bladders of mice with homozygous mutations of either *Lrig2* or *Hpse2*. The major nerve trunks within the normal murine bladder body contain cholinergic parasympathetic neurons that stimulate detrusor smooth muscle contraction.^3^ This feature may correlate with the detrusor overactivity typical of patients with UFS^4^ and that also is reported to be present in *Hpse2* homozygous mutant mice.^12^ As with the long-term changes in the walls of *Lrig2*^*–/–*^ bladders, increased nerve density in the bladder body may be secondary to chronic outlet obstruction. On the other hand, loss of Lrig2 can, in some circumstances, directly cause neuronal overgrowth, and Lrig2 knockdown causes overexuberant regeneration after optic nerve injury.^31^

We note that molecules involved in nerve biology are dysregulated in bladders of newborn *Lrig2* mutant mice. Upregulated transcripts included *Wnt family member 4*, encoding a growth factor implicated in establishing motor neurons^39^ and neuromuscular junctions^40^, ^41^; *period circadian regulator 2*, encoding period circadian clock 2, implicated in micturition rhythm and expressed in bladders^42^; and *shroom family member 1*, a member of a family implicated in central nervous system morphogenesis.^43^ Apart from *Lrig2*, downregulated transcripts included *G protein–coupled receptor 17*, encoding a leukotriene-responsive G protein–coupled receptor implicated in neural damage^44^ and glial maturation^45^; *capping actin protein of muscle Z-line subunit alpha 1*, encoding an F-actin–capping protein involved in neurite extension^46^; *Wnt family member 2B* that maintains a neural progenitor state in retinal progenitors during embryogenesis^47^; *contactin associated protein like 2,* encoding a presynaptic protein implicated in neuronal migration^48^; and *SRY-box 10*, encoding a transcription factor expressed in neural crest progenitors and glia in autonomic ganglia.^21^, ^25^, ^49^ Further experiments are needed to define whether these molecules are functionally implicated in UFS. Lrig2 binds neogenin, a receptor for neuronal guidance molecules, and prevents neogenin shedding mediated by ADAM metallopeptidase domain 17–mediated proteolysis.^31^ Transcripts encoding neogenin and ADAM metallopeptidase domain 17 were unchanged in the *Lrig2*^*–/–*^ neonatal bladder transcriptome.

Biallelic putative null variants of *LRIG2* have been reported in a subset of families with UFS.^13^ Further emphasizing the role for Lrig2 in bladder biology, this study discovered novel homozygous missense *LRIG2* variants in people with nonsyndromic bladder outlet obstruction. The collective evidence we have presented suggests that each variant is likely to be pathogenic. In the future, proof of pathogenicity could be provided by finding dysfunctional bladders in mutant mice engineered to be homozygous for either variant. Neither patient had facial features of UFS, something also noted in a case report of a child^13^ with bladder disease harboring a homozygous *LRIG2* variant (c.2125C>T) resulting in a stop codon. Therefore, evidence exists that *LRIG2* variants occur in some persons with nonsyndromic bladder outlet obstruction. Conversely, we found no strong evidence that *LRIG2* variants are implicated in primary nonsyndromic VUR, and we reached a similar conclusions regarding *HPSE2* in this condition.^10^ Congenital bladder voiding dysfunction can be also caused by variants of *acta alpha 2, smooth muscle* encoding *alpha smooth muscle actin*,^50^ *acta gamma 2, smooth muscle* encoding γ2-actin,^51^ *cholinergic receptor muscarinic 3* encoding an acetylcholine receptor,^52^ *myosin heavy chain 11*, encoding myosin heavy chain 11,^53^ and *myosin light chain kinase*, encoding a kinase involved in myosin activation.^54^ Whether any of these are implicated in primary nonsyndromic VUR awaits investigation.

## Materials and Methods

### Animals

Experiments were approved by the University of Manchester Biological Services Facility Committee and the United Kingdom Home Office (PPL 40/3550 and PAFFC1ffF), along with the Regional Ethics Committee of Umeå University (A193-12 and A1-2016). Experiments were conducted using Animal Research: Reporting of *In Vivo* Experiments best practice guidelines. *Hpse2* mutant mice studied here were created by gene-trap insertion into intron 6.^10^

### *Lrig2* mouse genotyping, VSOP, qRT-PCR, *in situ* hybridization, RNA sequencing, Western blot, whole bladder sheet processing, pelvic ganglia explants, and statistical analyses

See Supplementary File.

### Human analyses

Informed consent or assent was obtained from subjects or their parents. Studies were approved by institutional ethics committees (University of Manchester [06138] and National Health Service [06/Q1406/52 and 11/NW/0021]). United Kingdom VUR DNA samples were collected under National Research Ethics Service number MREC/01/6/15, with the clinical cohort previously published.^26^, ^55^ The 4C study of children with chronic renal failure has been described (http://www.4c-study.org/). *LRIG2* was analyzed using Sanger sequencing.

## Disclosure

All the authors declared no competing interests.

## Figures and Tables

**Figure S6:**
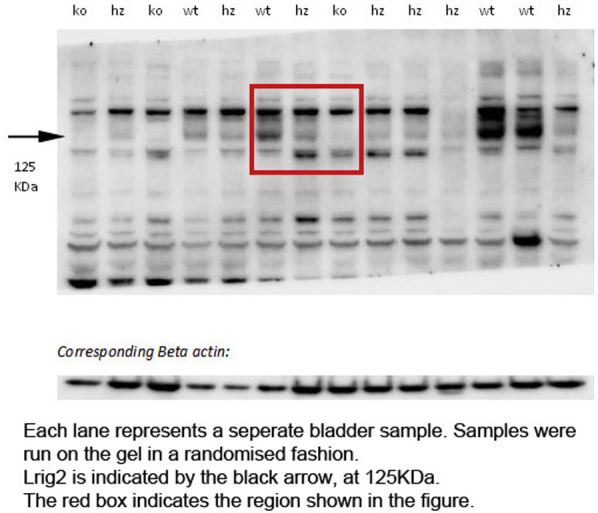
Western blot quantification of Lrig2 protein levels in bladder lysates. Total protein was extracted from wild-type, *Lrig2* heterozygous, and *Lrig2* homozygous mutant mouse bladders and 35 μg resolved on a polyacrylamide gel. Each lane represents a separate bladder sample, with genotypes loaded at random. Wild-type Lrig2 is indicated by the black arrow, at 125 KDa. The red box indicates an example of wild-type, heterozygous, and homozygous bladder samples, with strong, low, and absent Lrig2 bands, respectively.

## Supplementary Material

Supplementary Results Modeling effects of leucine-rich repeats and Ig-like domains 2 variants.

Supplementary Methods

Supplementary References
