# Supplementary material for: Lrig2 and Hpse2, mutated in urofacial syndrome, pattern nerves in the urinary bladder
Source: Kidney Int. 2019 May;95(5):1138–52. doi: 10.1016/j.kint.2018.11.040 (PMC6481288; doi:10.1016/j.kint.2018.11.040)
Supplement: Supplementary Results — Modeling effects of leucine-rich repeats and Ig-like domains 2 variants. [file mmc1.docx]

**SUPPLEMENTARY FILE**

**Lrig2 and Hpse2, mutated in urofacial syndrome, pattern nerves in the urinary bladder**

Neil A. Roberts^1^, Emma N. Hilton^1^, Filipa M. Lopes^1^, Subir Singh^1^, Michael J. Randles^2^, Natalie J. Gardiner^3^, Karl Chopra^1^, Riccardo Coletta^1,4^, Zunera Bajwa^1^, Robert J. Hall^5,6^, Wyatt W. Yue^7^, Franz Schaefer^8^, Stefanie Weber^9^, Roger Henriksson^10,11^, Helen M. Stuart^5,6^, Håkan Hedman^10^, William G. Newman^5,6^, and Adrian S. Woolf^1,4^.

^1^Division of Cell Matrix Biology and Regenerative Medicine, School of Biological Sciences, Faculty of Biology Medicine and Health, University of Manchester, UK.

^2^School of Allied Health Sciences, De Montfort University, Leicester, UK.

^3^[Division of Diabetes, Endocrinology and Gastroenterology](https://www.research.manchester.ac.uk/portal/en/facultiesandschools/division-of-diabetes-endocrinology--gastroenterology(e955d843-88a3-48d9-9ee5-131d97337a41).html), School of Medical Sciences, Faculty of Biology, Medicine and Health, University of Manchester, Manchester, UK.

^4^Royal Manchester Children’s Hospital, Manchester University NHS Foundation Trust, Manchester Academic Health Science Centre, Manchester, UK.

^5^Division of Evolution and Genomic Sciences, School of Biological Sciences, Faculty of Biology, Medicine and Health, University of Manchester, UK.

^6^Manchester Centre for Genomic Medicine, St. Mary’s Hospital, Manchester University NHS Foundation Trust, Manchester Academic Health Science Centre, Manchester, UK.

^7^Structural Genomics Consortium, Nuffield Department of Clinical Medicine, University of Oxford, UK.

^8^Division of Paediatric Nephrology, Centre for Paediatric and Adolescent Medicine, University Hospital of Heidelberg, Im Neuenheimer Feld, Heidelberg, Germany.

^9^Pediatric Nephrology, University-Children's Hospital Marburg, Philipps-University Marburg, Germany.

^10^Department of Radiation Sciences, Oncology, Umeå University, Umeå, Sweden

^11^Regional Cancer Center Stockholm/Gotland, Stockholm, Sweden.

**SUPPLEMENTARY RESULTS**

**Modelling effects of LRIG2 variants**

While human LRIG2 structure is not available, the extracellular LRR and IgL domains of human LRIG1 are structurally characterized (Xu et al 2015) and bear an approximate 50% sequence similarity to LRIG2 (Figure 8b). The two homozygous missense *LRIG2* variants described may impact on the structural integrity of the LRIG2 extracellular region by affecting disulphide bonds for Leu446Pro (Figure 8c) and a salt bridge for Arg650Cys (Figure 8d). Leu446 is positioned between the last LRR and first IgL domain, a region enriched with four conserved cysteines forming two intra-molecular disulphide bonds, important for extracellular domain orientation. Leu446 immediately precedes Cys447, one of the four disulphide-bonded cysteines. This amino acid position is highly conserved among LRIG1/2/3, and other LRR proteins. Therefore, substitution of Leu446 by Pro likely alters the local structural arrangement of Cys447 and Cys449, possibly impacting on disulphide bond formation. Arg650 is located within the second IgL domain. Sequence in the IgL domain is more variable evolutionarily, although this Arg residue and a downstream Asp residue (Asp669) are well conserved among each of the three IgL modules in LRIG2, and across the LRIG1/2/3 proteins. In the homologous LRIG1 structure, the conserved Arg and Asp pair form a salt bridge linking two neighbouring beta strands in the Ig fold, an interaction likely to be conserved in LRIG2. Hence the Arg650Cys variant will likely disrupt this salt bridge.
