# Supplementary material for: Lrig2 and Hpse2, mutated in urofacial syndrome, pattern nerves in the urinary bladder
Source: Kidney Int. 2019 May;95(5):1138–52. doi: 10.1016/j.kint.2018.11.040 (PMC6481288; doi:10.1016/j.kint.2018.11.040)
Supplement: Supplementary Methods [file mmc2.docx]

**SUPPLEMENTARY MATERIALS AND METHODS**

***Lrig2* mouse genotyping**

Genotyping primers for *Lrig2* mutant mice were: *Lrig2E12*-ablated, 5’ TGGGAGTGAGCTAGGCAG; for *Lrig2*-wild-type, 5’ TGCACTAGGCAGTCTTAAACCA; and for *Lrig2* common for ablated and wild-type, 3’ TCAGGCAGTGACAGAAGGTGTG.

**VSOP**

Mice were placed separately in cages with folded Whatman Grade 1 filter paper fixed to the bottom of the cage for three hours. The filter paper was removed from the cage, brushed to clean, and air dried for one hour. Filter paper was imaged under UV in a ChemiDoc (Leica) with 0.5 second exposure on a 26 cm by 19 cm field. Urine spots were quantified by ImageJ. The volume of each spot was calculated by comparing it to the pixel area of known volumes of urine (1 μl to 100 μl) applied to filter paper, deriving the function in excel and applying to the pixel area of the VSOP urine spots.

**RNA sequencing**

For the RNA sequencing we used female littermates. Whole neonatal bladders were homogenized using Biopulveriser Lysing Matrix D beads on a Fast-Prep-24 (MP Biomedicals). RNA was isolated using the Qiagen RNeasy kit. RNA-Seq libraries were generated using the SOLiD^™^ Total RNA-Seq Kit. Samples were run on SOLiD^™^ v4 for single-end 50 bp reads. The fastq files generated by HiSeq were analysed with FastQC (<http://www.bioinformatics.babraham.ac.uk/projects/fastqc/>) and any low quality reads and contaminated barcodes are trimmed with Trimmomactic (Bolger et al 2014). Libraries were aligned to mm10 assembly of mouse genome using Tophat-2.1.0 (Kim et al 2013) and matches with the best score were reported for each read. Mapped reads were counted by genes with HTSeq (Anders et al 2015) against gencode.vM2.annotation.gtf. Principle component analysis of all detected transcripts was performed using MATLAB. Differentially expressed genes were identified with an R package DESeq2 (Robinson et al 2010; Love et al 2014). Z-transformed mean normalized intensities were used for hierarchical clustering of the transcript data. Agglomerative hierarchical clustering was performed and visualized using MultiExperiment Viewer (version 4.8.1), and gene clusters were analyzed using DAVID GOEA and Gene Ontology Consortium using Panther classification (<http://www.geneontology.org/>).

**qRT-PCR**

RNA was extracted as described in the RNA sequencing, and 1 μg used to generate cDNA using the High-Capacity RNA-to-cDNA kit (Applied Biosystems). cDNA was diluted to a 1 / 20 working concentration and 20ul reactions were performed using 1 ul of cDNA working stock. Reactions were performed using Taqman Gene Expression MasterMix (Applied Biosystems) on a StepOnePlus platform (Applied Biosystems) and data analyzed using the StepOne software and rendered using PRISM. Taqman assay IDs used were: *Hprt* Mm03024075_m1 (housekeeper); exon 12-13 *Lrig2* Mm01305500_m1; exon 17-18; *Lrig2* Mm01305504_m1. *Nos1* primers were obtained from Applied Biosystems (Mm01208059_m1).

***In situ* hybridization**

E15 mouse bladders were fixed overnight in 4% PFA and in situ hybridisation was performed as previously described (Ridge et al 2017). Primers used to generate probes were T7: ATTGGCCTTGAGTCCCTTGA and T3: CACTGTCCTTGCGCCATATG, spanning *Lrig2* exons 11 to 13. Sense and Antisense probes were synthesized from PCR linear template DNA product by using RNA polymerase, digoxygenin nucleotide mix and transcription buffer.

**Antibodies**

For western blotting, we used rabbit anti-Lrig2 (3 μg/ml, mLrig2-147),^18^ overnight at 4^o^C. For immunohistochemistry, we used protocols followed as described.^9^ Primary antibodies used were: mouse anti-heparanase (1:200, Serotec OBT1975G); rabbit-anti heparanase 2 (1:200, custom-designed to epitope QLDPSIIHDGWLD); rabbit anti-Lrig2 (1:400 mLrig2-147 and Abgent AP13821b); rabbit anti-Ki67 (1:500, Abcam, Ab16667); rabbit anti-Lrig2 (1:400 mLrig2-147 and Abgent AP13821b); goat anti-nNOS (1:500, R&D, AF2416); goat-anti uroplakin II (1:500, Santa Cruz, sc-15178); and rabbit anti-Wnt4 (1:200, Abcam ab91226). Biotinylated secondary antibodies were anti-rabbit (1:200, Sigma B8895) and anti-goat (1:200, Vector Laboratories, BA-9500). Detection with biotinylated secondary antibodies: anti-rabbit (1:200, Sigma B8895) and anti-goat (1:200, Vector Laboratories, BA-9500). For immunostaining of whole bladder sheets, the following were used: goat anti-nNOS (1.200, R&D, AF2416); rabbit anti-Peripherin (1:300, Merck Millipore, AB1530); and chicken anti-TH (1:200, Abcam, AB76442). Detection with Alexafluor secondary antibodies (Thermo Fisher Scientific) at 1:300: donkey anti-rabbit 568; donkey anti-goat 594; goat anti-chicken 488. For immunocytochemistry, primary antibodies used were: anti heparanase 2 and anti-Lrig2, as above; chicken anti-β3-tubulin (1:500, Merck Millipore NG1809690); and mouse anti-S100 (Sigma 2532).

**Western blot**

Each bladder was homogenized in a RIPA extraction buffer including protease inhibitors by mechanical disruption using a Precellys24 homogenizer (Saveen & Werner AB, Limhamn, Sweden). Protein was cleared by centrifugation at 20,800 x g for 15 minutes at 4°C and denatured in NuPAGE-LDS sample buffer (NP0007, Thermo Fisher Scientific, Gothenburg, Sweden) supplemented with NuPAGE sample reducing agent (NP0009, Thermo Fisher Scientific) and heated at 95^o^C for 5 minutes. Thirty five μg (for Lrig2) or 2 μg (for β-actin) of protein was loaded per lane and membranes blocked in 5 % milk powder in PBSTween 0.1 % before incubation with rabbit anti-Lrig2 (3 μg/ml, mLrig2-147, Rondahl 2013) or β-actin antibody overnight at 4^o^C. Primary antibodies were detected with an anti-rabbit antibody and an HRP based colour reaction. Bands were quantified in ImageJ.

**Whole bladder sheet processing and immunostaining**

Bladders from mice aged one to two weeks were excised and washed in PBS then fixed whole or opened ventrally (to preserve the pelvic ganglia and trigone) and fixed flat between glass slides to create bladder sheets, in 4 % paraformaldehyde (Sigma) at 4^o^C for 24 hours. Bladders were stored in methanol (MeOH). Prior to staining, bladders were post-fixed in Dent’s fixative (1:1:4, 30% H2O2:DMSO:MeOH) overnight at 4^o^C then washed in MeOH three times and rehydrated to PBS. Bladders were washed in PBS-0.2% Tween-100mg/ml heparin (PTwH) for 30 minutes, then blocked in PBS-0.1% Triton X-1% BSA (PxB) for one hour, then PxB-5% donkey serum (PxBD) for two hours. Primary antibody was added to the samples in PxBD-5% DMSO-sodium azide, in 600ml volume, and incubated at room temperature over two nights. Primary antibodies were rabbit anti-peripherin (1:300, Merck Millipore, AB1530). Samples were washed in PxB for an hour and further washed in PTw with at least three changes and overnight at 4^o^C. The next day samples were blocked in PxB and PxBD (one and two hours respectively) and secondary antibody (donkey anti-rabbit 568, Life Technologies, A11034) was added in in PxDB-sodium azide, in the dark at 4^o^C, for 24 hours. Samples were washed in PxB followed by Pw for at least four changes, including overnight 4^o^C incubation, then dehydrated to MeOH and stored until imaging. For imaging, samples were cleared in 1:2 benzyl alcohol:benzyl benzoate (Murray’s clearing medium) and transferred to a glass bottomed culture dish. Initial *Lrig2* bladders were imaged on an LSM880 (Zeiss) Airyscan on a 2.5x objective with 1.5x digital backzoom to allow capture of the whole bladder. Subsequent experiments, including the *Hpse2* bladders, were imaged on an Axio Observer.Z1 LED (Zeiss) on a 10x objective to reduce bleaching and allow better detail. Images were compared blindly by four individuals by counting the number of neuronal crossings of three lines, giving an output of neuronal density. For nerve density quantification, the three-line grid was positioned in Photoshop above the pelvic ganglia in the bladder body. The large trunk nerves were used as landmarks to consistently position the lines in the horizontal plane, and distinctive neuronal ‘arches’ observed in the bladder body were used to position line in the vertical plane.

**Pelvic ganglia explants**

Pelvic ganglia from multiple E14 embryos from the same litter were dissected, pooled and minced using an Optimum Straight Knife (BD Beaver, 370566).  Pelvic ganglia were then suspended in culture media: DMEM/F12 (Sigma, D8437) with 20 ng/ml NGF (Sigma, N6009) and penicillin G 100μ/ml, streptomycin 100 μg/mL and amphotericin B 0.25 μg/ml (Sigma, A5955) then plated on laminin (Sigma, L2020) coated 8-chamber slide (Falcon, 354108) and incubated for 48 hours at 37°C in 5% CO_2_. Prior to plating, 8-chamber slides were coated with laminin 2 μg/ml in PBS with calcium chloride and magnesium chloride (Sigma, D8662) for 45 minutes and then washed three times with PBS with calcium chloride and magnesium chloride. Slides were fixed in 2% paraformaldehyde (Sigma, P6418) for 20 minutes and washed 3 times in PBS (Sigma, D1408). Primary antibodies in PBS 0.2% Triton X-100 (Sigma, T8787) 5% donkey serum (Sigma, D9663) was added to slides and incubated overnight in a dark humidity chamber at 4°C. Primary antibodies used were: mouse anti-heparanase (Serotec OBT1975G); goat anti-heparanase 2 (1.250, Santa Cruz AB97807); rabbit anti-Lrig2 (1.400, mLrig2-147 and Abgent AP13821b); mouse anti-S100B (1.500, Sigma, S2532); Slides were then washed 3 times in PBS. Secondary antibodies in PBS 0.2% Triton X-100 and 5% donkey serum were added to slides and incubated for 30 minutes. Secondary antibodies were used at 1.500 (Life technologies, anti-mouse 488, A10667; anti-rabbit 594, A11034; anti-goat 594, A11058; anti-chicken 488, A11042). Slides were removed from the chamber mount and washed three times in PBS, three times in H_2_O and the left to dry overnight. Once dry, vectorshield mounting medium with DAPI (Vector Labs, H1200) was added to slides and then slides mounted and sealed with nail varnish. Phase contrast images of pelvic ganglia growth were captured on an Olympus IX51 inverted microscope using 20x and 40x objective lens (Olympus, Lcach) and captured using a QIClick camera (QImaging) and Q-Capture Pro7 software. Images underwent post-capture processing in Fiji and Photoshop. Immunofluorescent images were captured on an upright microscope (Olympus BX51) using objective lens, 40x and 60x (UPlanFLN) and captured using a Coolsnap ES camera and Metavue software. Filters for DAPI, FITC and Texas red were used to prevent bleed through. Images underwent post-capture processing in Fiji and Photoshop.

**Statistical analyses**

Statistical tests were performed using PRISM to compare control and Lrig2^-/-^ genotypes, unless stated otherwise. For qRT-PCR, *Lrig2* transcript levels were tested for normal distribution using the Shapiro-Wilk normality test then compared using 2 tailed unpaired Student’s t-test. For western blot, Lrig2 band intensity in wild-type, heterozygous and homozygous *Lrig2* knockout bladders was tested for normal distribution using the Shapiro-Wilk normality test then compared using ordinary one-way ANOVA with Tukey’s multiple comparisons test. For the bladder necropsy the Fisher’s exact test for small numbers was used to compare the number of empty and containing-urine bladders. For the VSOP urine analysis 2 tailed unpaired Student’s t-test was used to compare urine spot number and area. Ki67 positive cells were compared using Student’s t-tests. For the bladder body nerve quantification the number of counts were tested for normality using the Shapiro-Wilk normality test then compared using unpaired Student’s t-test. nNOS immunohistochemical semi-quantification and *Nos1* transcript quantification were compared littermate pairs using paired Student’s t-tests.
