# Supplementary material for: Lrig2 and Hpse2, mutated in urofacial syndrome, pattern nerves in the urinary bladder
Source: Kidney Int. 2019 May;95(5):1138–52. doi: 10.1016/j.kint.2018.11.040 (PMC6481288; doi:10.1016/j.kint.2018.11.040)
Supplement: Supplementary References [file mmc3.docx]

**SUPPLEMENTARY REFERENCES**

Xu Y, Soo P, Walker F, Zhang HH et al. LRIG1 extracellular domain: structure and function analysis. *J Mol Biol* 2015;427:1934-1948.

[Anders S](https://www.ncbi.nlm.nih.gov/pubmed/?term=Anders%20S%5BAuthor%5D&cauthor=true&cauthor_uid=25260700), [Pyl PT](https://www.ncbi.nlm.nih.gov/pubmed/?term=Pyl%20PT%5BAuthor%5D&cauthor=true&cauthor_uid=25260700), [Huber W](https://www.ncbi.nlm.nih.gov/pubmed/?term=Huber%20W%5BAuthor%5D&cauthor=true&cauthor_uid=25260700). HTSeq--a Python framework to work with high-throughput sequencing data. *Bioinformatics* 2015;31:166-169.

Bolger AM, Lohse M, Usadel B. Trimmomatic: a flexible trimmer for Illumina sequence data. *Bioinformatics* 2014;30:2114-2120.

[Kim D](https://www.ncbi.nlm.nih.gov/pubmed/?term=Kim%20D%5BAuthor%5D&cauthor=true&cauthor_uid=23618408), [Pertea G](https://www.ncbi.nlm.nih.gov/pubmed/?term=Pertea%20G%5BAuthor%5D&cauthor=true&cauthor_uid=23618408), [Trapnell C](https://www.ncbi.nlm.nih.gov/pubmed/?term=Trapnell%20C%5BAuthor%5D&cauthor=true&cauthor_uid=23618408) et al. TopHat2: accurate alignment of transcriptomes in the presence of insertions, deletions and gene fusions. *Genome Biol* 2013;14:R36.

[Love MI](https://www.ncbi.nlm.nih.gov/pubmed/?term=Love%20MI%5BAuthor%5D&cauthor=true&cauthor_uid=25516281), [Huber W](https://www.ncbi.nlm.nih.gov/pubmed/?term=Huber%20W%5BAuthor%5D&cauthor=true&cauthor_uid=25516281), [Anders S](https://www.ncbi.nlm.nih.gov/pubmed/?term=Anders%20S%5BAuthor%5D&cauthor=true&cauthor_uid=25516281). Moderated estimation of fold change and dispersion for RNA-seq data with DESeq2. *Genome Biol* 2014;15:550.

Ridge LA, Mitchell K, Al-Anbaki A et al. [Non-muscle myosin IIB (Myh10) is required for epicardial function and coronary vessel formation during mammalian development.](https://www.ncbi.nlm.nih.gov/pubmed/29084269) *PLoS Genet*. 2017;13:e1007068

[Robinson MD](https://www.ncbi.nlm.nih.gov/pubmed/?term=Robinson%20MD%5BAuthor%5D&cauthor=true&cauthor_uid=19910308), [McCarthy DJ](https://www.ncbi.nlm.nih.gov/pubmed/?term=McCarthy%20DJ%5BAuthor%5D&cauthor=true&cauthor_uid=19910308), [Smyth GK](https://www.ncbi.nlm.nih.gov/pubmed/?term=Smyth%20GK%5BAuthor%5D&cauthor=true&cauthor_uid=19910308). edgeR: a Bioconductor package for differential expression analysis of digital gene expression data. *Bioinformatics* 2010;26:139-140.
